# Integrative Prevention at Work: Protocol for the Development of an Assessment Tool

**DOI:** 10.2196/98362

**Published:** 2026-11-03

**Authors:** Andrée-Anne Drolet, Alexandra Lecours, Claude Vincent, Marie-Eve Major, Valérie Lederer, Christian Longtin

**Affiliations:** 1 Département d'ergothérapie, Université du Québec à Trois-Rivières, 3351, boulevard des Forges, Trois-Rivières, QC, G9A 5H7, Canada, 1 819 376-5011 ext 2936; 2 Centre interdisciplinaire de recherche en réadaptation et intégration sociale, Québec, QC, Canada; 3 École des sciences de la réadaptation, Université Laval, Québec, QC, Canada; 4 Département des relations industrielles, Université Laval, Québec, QC, Canada; 5 Département des relations industrielles, Université du Québec en Outaouais, Gatineau, QC, Canada; 6 École de réadaptation, Université de Sherbrooke, Longueuil, QC, Canada

**Keywords:** integrative prevention at work, occupational health, disability management, instrument development, validation

## Abstract

**Background:**

In a changing world of work, integrative prevention at work represents a promising avenue for addressing contemporary health, safety, and well-being issues. However, for organizations to deploy and benefit from this approach, developing an assessment tool for integrative prevention appears to be the first required step. No existing assessment tool can assess all its key characteristics on a unified scale, limiting the operationalization of this approach in organizations.

**Objective:**

This study aims to develop an assessment tool to evaluate the 5 attributes of integrative prevention at work within the health and social services sector. Specifically, the study aims to: (1) generate items, rating scales, and instructions; (2) validate the content of the tool; (3) pretest the tool in organizational settings; and (4) evaluate its psychometric properties.

**Methods:**

A 4-phase methodological study will be carried out for each research objective. The assessment tool will be developed and tested in health and social services organizations in Quebec, Canada.

**Results:**

Phase 1 is complete, with a total of 96 items created and distributed relatively evenly across 5 subscales, each reflecting one attribute of integrative prevention at work. Phase 2 will be finalized by the end of 2026. Phases 3 and 4 will be completed by the end of 2028 to provide a validated tool to assess the attributes of integrative prevention at work.

**Conclusions:**

From an organizational perspective, this tool will provide health and social services organizations with a validated, context-adapted measure of the attributes of integrative prevention at work, enabling baseline assessment, targeted improvements, and longitudinal monitoring, thereby strengthening prevention practices. From a research perspective, this project will deepen the understanding of integrative prevention at work through its empirical validation.

## Introduction

### Background

Work is a well-established determinant of health [1,2] and can positively influence individuals’ health and well-being when performed under favorable conditions [3]. In addition to providing financial security, work can offer social recognition, meaningful social connections, and protection against the decline of certain functional capacities [4-6]. Conversely, adverse working conditions and occupational injuries, whether physical or psychological, can undermine quality of life and reduce participation in the labor force [7]. Beyond their consequences for individuals, occupational injuries also have organizational repercussions, including increased absenteeism [8] and decreased workplace performance [9]. For over 3 decades, the scientific community and various stakeholders (eg, work organizations, governments, and health care professionals) have devoted sustained attention to preventing occupational injuries [10,11]. Yet, prevention remains a highly relevant issue due to the emergence of new challenges in today’s evolving world of work [12-14]. Indeed, contemporary work environments are being reshaped by structural changes such as labor shortages, pandemic-related disruptions, the rapid rise of telework, and the integration of AI [15-17]. These transformations have altered how work is performed and may heighten occupational risks (eg, increased workload due to staff shortages, anxiety related to fears of technological replacement). They also complicate the management of risk consequences, particularly the adaptation of return-to-work processes following periods of absence [17-19]. Traditional prevention strategies, while valuable, often fall short in addressing these emerging challenges [15,19-21]. Organizations therefore stand to benefit from innovative prevention approaches to sustain workers’ health and well-being. In this context, the paper outlines a study protocol for the development of an assessment tool to support organizations in adopting a new prevention approach known as integrative prevention at work.

### State of Knowledge

The need for more effective prevention approaches becomes even more pressing in light of the rising number of occupational injuries observed globally. In the province of Quebec (Canada), the Workers’ Compensation Board reported a 56.6% increase in occupational injury cases between 2018 and 2022, despite the numerous prevention efforts already implemented in workplaces [22]. In the United States, the Bureau of Labor Statistics recorded 2.8 million nonfatal injuries and illnesses in the private sector in 2022, an increase of 7.5% compared to 2021 [23]. A similar trend was observed in the European Union, where in 2021, 2.88 million nonfatal work accidents resulting in at least 4 calendar days of absence were reported, representing a 5.5% increase from the previous year [24]. These figures highlight the need to adapt and improve preventive actions to reduce both the incidence and consequences of occupational injuries. The importance of improved prevention is further underscored by the human costs such as the pain and distress experienced by injured workers [25]. In Quebec, human costs represent 61% of the total burden associated with occupational injuries [25,26]. The financial repercussions are also considerable. In the United States, occupational injuries cost an estimated US $167 billion in 2021 [27]. As for the European Union, the estimated annual cost of work-related accidents and illnesses reaches approximately €476 billion (US $534.7 billion) [28]. Similarly high costs are reported in other industrialized countries such as Australia (Aus $28.60 billion [US $20.10 billion]) [29] and Great Britain (£20.70 billion [US $27.70 billion]) [30]. Currency conversions were calculated using the European Central Bank reference exchange rates published on October 21, 2026: €1=US $1.12, Aus $1=US $0.70, and £1=US $1.34. These statistics demonstrate that the consequences of occupational injuries extend far beyond individual workers, impacting organizations and society as a whole.

### Continuum of Prevention for Occupational Injuries and Prolonged Incapacity at Work

Traditionally, the continuum of prevention for occupational injuries and prolonged incapacity at work has been structured along 3 levels: primary, secondary, and tertiary prevention [31]. Primary prevention concerns actions that aim to prevent the occurrence of an occupational injury. These interventions often involve modifications to the work environment to reduce risks and hazards, thereby benefiting the entire workforce [32-34]. Secondary prevention aims to halt or delay the progression of an occupational injury and to mitigate its consequences. This level primarily targets individual workers and includes actions such as early detection and intervention following exposure [32-34]. Tertiary prevention aims to minimize the risk of relapse and chronicity after an occupational injury [32]. Interventions at this level include rehabilitation, effective return-to-work processes, and strategies to prevent recurrences or the deterioration of workers’ health [32,33]. Some authors also suggest considering health promotion as a part of this continuum [35,36]. Such initiatives aim to empower workers to take greater control over their health and well-being, for example, by providing healthy food options in workplace cafeterias or by creating supportive environments that foster an active lifestyle. Occupational health prevention and promotion requires the involvement of multiple stakeholders, including those from the health care system (eg, rehabilitation professionals, nurses, physicians, and ergonomists), the workplace (eg, managers, workers, and unions), and the insurance sector (eg, public or private insurers) [37-39]. While valuable, these approaches often remain fragmented, with limited coordination across stakeholders [37,38]. As a result, several scholars have called for a more comprehensive and integrated approach that would benefit both organizations and workers [13,32,40,41].

### Conceptualization of Integrative Prevention at Work

In recent years, integrative prevention at work has emerged as a promising and contemporary avenue to address the prevention of occupational injuries and prolonged incapacity at work. This approach is grounded in a philosophy that promotes the coordination of prevention actions among stakeholders and the removal of boundaries between the traditionally distinct levels of prevention (ie, primary, secondary, and tertiary) [32,33,40-43]. This holistic approach has attracted growing interest across various sectors, including the scientific community [44], due to its potential to produce significant positive impacts, not only for workers, but also for organizations and society at large, including financial benefits [45-47]. To clarify the concept of integrative prevention at work and identify its operational characteristics, our team conducted a research project divided into 2 phases. In the first phase, we carried out a concept analysis following Walker and Avant’s method [48], using a meta-narrative review to examine publications addressing the integration of prevention activities across the prevention continuum for occupational injuries and prolonged incapacity at work [41]. This 5-step systematic process involved identifying, extracting, and synthesizing information on the attributes, antecedents, outcomes, and contextual factors of integrative prevention at work across the literature, which were used to develop a preliminary conceptualization. In the second phase, we collected qualitative data based on the experiential knowledge of key stakeholders involved in the prevention continuum for occupational injuries and prolonged incapacity at work. This qualitative phase aimed to enrich and refine the preliminary conceptualization, ensuring that it more accurately reflects organizational realities and practice settings [49]. The findings from both phases enabled us to propose a comprehensive conceptualization of integrative prevention at work, illustrated in Figure 1.

**Figure 1.**
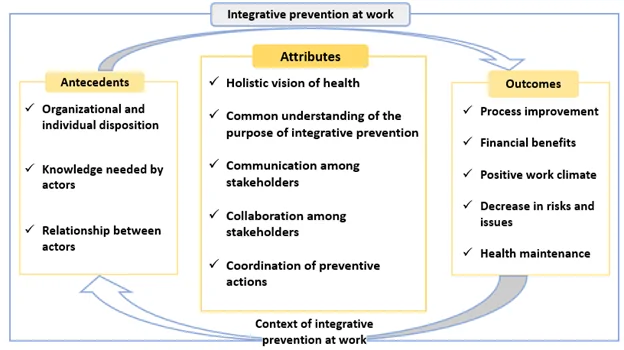
Attributes, antecedents, outcomes, and contextual factors related to integrative prevention at work, adapted from Bellehumeur-Béchamp et al [49].

According to our findings, integrative prevention at work is characterized by the actualization of 5 key characteristics, referred to as attributes (ie, the manifestation of the approach in reality) [49]. These attributes are: (1) a holistic vision of health, considering workers’ physical and mental health, as well as their overall well-being, as interconnected dimensions; (2) a common understanding of the purpose of integrative prevention, referring to a shared view among stakeholders of what integrative prevention aims to achieve; (3) communication between stakeholders, involving the exchange of information, knowledge, and perspectives among all stakeholders; (4) collaboration between stakeholders, involving working together to address prevention needs by sharing expertise, responsibilities, and resources; and (5) coordination of preventive actions, aligning preventive actions across stakeholders and levels of prevention to ensure that they complement rather than conflict with one another. The implementation of these attributes within a work context could lead to outcomes (ie, characteristics that emerge from or are influenced by the approach) such as process improvement, financial benefits, positive work climate, decrease in risks and issues, and health maintenance [49]. However, antecedents (ie, characteristics that precede the approach) are required beforehand to be able to actualize attributes and generate outcomes. Organizations and individuals need to be disposed, stakeholders need knowledge, and a relationship must exist between them.

All attributes, antecedents, and outcomes must be implemented according to a particular context, which is part of an ongoing process of change [41,49]. In fact, integrative prevention at work can be seen as a dynamic and systemic process. More precisely, the attributes are closely connected and reinforce each other, helping to create an organizational culture of prevention. For example, communication without collaboration, or coordination without a shared vision, is unlikely to produce lasting effects. What matters is the way the attributes work together to embed prevention in everyday practices and relationships. This conceptualization of integrative prevention at work provides a clear and structured description of the approach, thus enabling its operationalization in real-world contexts [50]. Our work has also highlighted the need for practical means such as operational tools, to support the transformation of prevention practices [41]. Despite this growing interest, no existing assessment tool currently measures all 5 attributes of integrative prevention at work on a unified scale [51]. Developing such a tool could serve as a valuable resource for organizations interested in adopting this approach [51].

### Health and Social Services Sector: A Relevant Context for Integrative Prevention at Work

The adoption of integrative prevention at work would be particularly relevant in the health and social services sector, as it faces significant challenges related to occupational health and injury prevention. In Quebec (Canada), for instance, the number of occupational injury cases in this sector increased by 311% between 2019 and 2023 [52,53]. In 2022, nursing professionals accounted for nearly 10% of all occupational injuries across all sectors in the province, a figure that rose to 19% among support staff in the health and social services sector [52]. A similar trend is observed in the United States, where the education and health services sector recorded the highest number of nonfatal injuries and illnesses in 2021 (including COVID cases) [54]. Likewise, in Europe, the health and social care sector is recognized as a high-risk sector [55]. These statistics can be explained by the various challenges faced by health care workers in recent years such as the COVID-19 pandemic. In addition to the contemporary challenges affecting all workers in today’s evolving world of work, health care professionals are especially vulnerable to psychological risks such as burnout [56,57] and are more likely to experience anxiety and other mental health concerns [58,59]. They are also at higher risk of musculoskeletal injuries due to physically demanding tasks such as patient lifting and transfer activities [60]. Moreover, this sector faces significant staff turnover [61]. Given these challenges, the implementation of integrative prevention at work appears relevant in health care organizations. Furthermore, the health and social services sector has demonstrated openness to initiatives focused on occupational health and work disability management (eg, [62,63]). In recent years, several organizations in this sector in Canada and the United States have increasingly turned toward integrative prevention approaches. Many have participated in research projects aimed at maintaining and improving health, security, and well-being at work [64-66], illustrating their growing interest in and need for such approaches. For example, a program called Prevention and Early Active Return-to-Work Safely (PEARS) was implemented in a large Canadian hospital (in British Columbia). The goal of this pilot project was to integrate secondary prevention actions into an existing primary prevention program. These initiatives specifically targeted musculoskeletal injury prevention. Despite low worker participation in this pilot program, the results supported the idea that coordinating primary and secondary prevention efforts is more effective at reducing disability than implementing them separately [65,66]. In the United States, a total worker health (TWH) initiative called the live well/work well (LWWW) was implemented across a large health care organization, including hospitals and medical centers. A TWH approach is defined as “policies, programs, and practices that integrate protection from work-related safety and health hazards with promotion of injury and illness-prevention efforts to advance worker well-being” [67]. Implementation of the LWWW program in medical centers and hospitals led to several benefits reported by participants, including workers feeling that their health and safety were protected by their organization, as well as improvements in lifestyle scores over a 3-year period. Considering these examples of initiatives, mostly compatible with our conceptualization of integrative prevention at work, implementing such an approach could serve as a promising solution for organizations in the health and social services sector. An assessment tool could support such organizations in the early stages of implementation and provide continued benefits throughout the change process.

### Rationale for Developing an Assessment Tool for Integrative Prevention at Work

To gain a comprehensive overview of the assessment tools currently available, our research team conducted a scoping review to identify and describe tools assessing one or more attributes of integrative prevention at work [51]. However, none of them assess all 5 attributes using a unified scale, which poses a challenge for the operationalization of this approach in organizations [51]. To address this gap, it is necessary to develop an assessment tool that would encompass all the attributes of integrative prevention at work on a unified scale. Such a tool would facilitate the implementation of this innovative prevention approach across diverse organizational contexts. According to Pronk et al [68], emerging approaches like integrative prevention at work necessitate tools that can assess their defining characteristics and measure their level of implementation. Even before the implementation process begins, an assessment tool can be used to establish a baseline within an organization, which can then serve as a reference point throughout the implementation journey [68]. Beyond this initial purpose, the tool could also serve as a practical guide for stakeholders by supporting the progressive integration of the approach’s defining characteristics into their specific contexts [33] and by enabling the monitoring of implementation progress over time [47,69,70]. Additionally, interpreting the results of the assessment tool in light of the approach’s characteristics could help organizations determine future objectives [68,70] and identify necessary organizational changes [71]. In this way, the tool could contribute to a continuous improvement process by helping organizations identify areas for improvement, set priorities, and guide the ongoing development of prevention practices. Beyond measurement, an assessment tool could also foster dialogue and reinforce stakeholder engagement in the prevention process [72], all of which are key attributes of integrative prevention at work [41,49].

### Problem Statement

Given that organizations are continuously confronted with emerging challenges [12-14] and that traditional means of prevention are no longer sufficient to address these complex issues [15,19-21], organizations would benefit from adopting new approaches to maintain workers’ health and well-being, such as integrative prevention at work [32,33,40-42,73]. Although recent studies have identified the defining key characteristics of integrative prevention at work [41,49], no assessment tool currently exists to evaluate them within organizations. This gap represents a key operational challenge [41].

In the health and social services sector, where challenges related to occupational injuries and prolonged incapacity at work are particularly acute (eg, [60,74,75]), organizations have demonstrated a particular openness and responsiveness to initiatives aimed at improving health, safety, and well-being in the workplace (eg, [62,63]). Given these considerations, developing an assessment tool to support the assessment of the attributes of integrative prevention at work represents a promising solution to facilitate the implementation of this approach in this sector.

### Objectives

This project aims to develop an assessment tool for evaluating the attributes of integrative prevention at work within the health and social services sector. More specifically, the project aims to: (1) generate items, rating scales, and instructions; (2) validate the content of the tool; (3) pretest the tool in organizational settings; and (4) evaluate its psychometric properties.

## Methods

### Overview

This project will use a methodological study design to develop an assessment tool [76-78], and will be carried out in 4 distinct phases, as illustrated in Figure 2. Since an assessment tool must be developed with careful consideration for the language and cultural context of its intended users [76], the tool to assess the attributes of integrative prevention at work will initially be developed in French, given that most health and social services institutions in Quebec operate primarily in this language.

**Figure 2.**
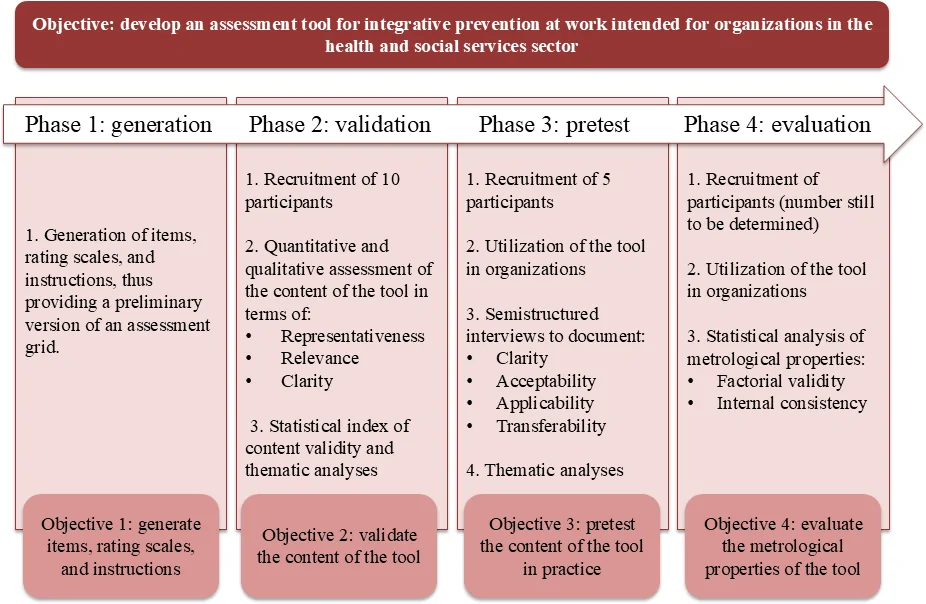
Methodology for the development of the assessment tool.

### Phase 1 – Generation

#### Procedure and Analysis

Before starting the development of the tool, we defined its purpose (ie, to assess the frequency with which attributes of integrative prevention at work are manifested in organizations within the health and social services organizations in Quebec) and the criteria to be measured (ie, the 5 attributes of integrative prevention at work) [76]. The attributes were chosen as criteria because they represent the key characteristics of the approach and, as such, should be assessed first when measuring an emerging approach [48]. It should also be noted that, although this tool assesses the frequency with which attributes of integrative prevention at work are manifested within a given organization, it will be completed individually by stakeholders based on their perception of the organizational practices and realities in the organization with which they work or with which they collaborate.

We developed our assessment tool based on a scoping review conducted by our research team [51]. As previously mentioned, no existing assessment tool captures all 5 attributes of integrative prevention at work within a single, unified measurement scale. The review identified twelve assessment tools. The initial item-generation process was led by the first author. Items were not directly transferred from the assessment tools identified in the scoping review. Rather, the findings gathered from these tools were used in an operationalization process, through which the extracted data were coded and categorized, and subsequently mapped onto the definition of the 5 attributes of integrative prevention at work [79]. The first author formulated the initial items based on this operationalization process, and the developing item pool was periodically discussed with the research team to assess its relevance, coherence, and alignment with the operational definition. This process resulted in the formulation of (1) observable items reflecting each attribute, (2) graded rating scales, and (3) standardized instructions [51,79]. In other words, information extracted from the identified tools was used to develop new items reflecting the operational definitions of the 5 attributes of integrative prevention at work, along with appropriate rating scales and standardized instructions. Accordingly, this phase allowed us to develop a preliminary version of the assessment tool based on existing literature in ergonomics, rehabilitation, human resource management, industrial relations, and organizational psychology. These domains were selected because they represent the principal fields in which integrative prevention at work and related approaches have been conceptualized and studied, and therefore provide the theoretical and empirical foundations for the development of the assessment tool [41,49]. The assessment tool was developed using an ordinal scale, with each subscale corresponding to one attribute, as this appeared to be a simple and effective method for assessing the attributes of integrative prevention [80]. A frequency scale was chosen to align with the objective of the assessment tool, which is to assess the frequency with which attributes of integrative prevention at work are manifested within an organization. This response format was considered appropriate because the 5 attributes are operationalized through observable organizational practices and actions that may occur with varying frequency. The dynamic nature of integrative prevention at work further supports this choice, as the occurrence of these practices may vary across situations and over time [79,81]. A common frequency scale ranging from “never” to “always” was selected for all items to facilitate comparisons across the 5 subscales and support the interpretation of results across attributes. For instance, if the score on the holistic vision of health subscale is lower than those of the other subscales, the organization can easily identify specific indicators related to this attribute to target for improvement. In other words, this could enable organizations to pinpoint specific characteristics of integrative prevention at work that require adjustments and implement appropriate changes.

Accordingly, all items were formulated to align with a frequency-based response scale. A 7-point ordinal scale was developed, as it provides enough response options to reflect meaningful variations in the frequency with which the attributes are manifested, while remaining cognitively accessible for its users [82,83]. Additionally, a 7-point scale is preferred when the target users are familiar with completing assessment tools and can differentiate between nuanced response options [83], which applies to this context. Indeed, the targeted users of this assessment tool are professionals who typically possess university-level education and are therefore more accustomed to reflective assessments related to organizational practices. This scale, along with the instructions and items that emerged from information collected through the scoping review, was submitted for validation.

### Phase 2 – Validation

#### Participants

To assess the content validity of the tool, 10 experts will be recruited [84-86] using a purposive sampling strategy. Inclusion criteria for the recruitment of the experts will be (1) having at least 2 years of expertise as a professional in a health and social services organization, or as a researcher, (2) being engaged in tasks, whether professional or research-related, linked to the prevention of occupational injuries or the support of prolonged incapacity at work, and (3) having experience related to prevention efforts aimed at workers in the health and social services sector (eg, ergonomists, human resources managers, and prevention specialists). Although it is recommended to recruit a minimum of 3 experts from each group (ie, professionals and researchers) [85], we aim to ensure a balanced representation by including approximately 5 participants per group. This target remains flexible and will depend on experts’ availability [84,85,87]. Also, while it is generally recommended to include at least one researcher with specific expertise in the development of assessment tools [85,87,88], our research team will include at least 2 such researchers, in order to strengthen the quality of the tool’s structure and design. The planned panel includes experts with diverse and complementary expertise to ensure that the assessment tool is both conceptually sound and practically relevant, while providing the breadth of knowledge required to adequately evaluate its content validity [84,88]. The sample size is also aligned with recommendations for content validation studies, which suggest that a relatively small panel of carefully selected experts is sufficient to establish content validity [85,87,88].

#### Procedure

The experts will receive a personalized email containing (1) a summary of the operational definition of integrative prevention at work, (2) the first version of the assessment tool, and (3) the questionnaire that will guide the validation process [82,89]. Experts will be asked to evaluate each item in terms of (1) representativeness of the content, (2) relevance to the concept, and (3) clarity of the formulation. These evaluations will be rated using a 4-point ordinal agreement scale [84]. For example, experts will be asked to address communication among stakeholders through the evaluation of items such as “Stakeholders transmit the necessary information efficiently to those who need it.” They will rate this item’s representativeness, relevance, and clarity on a 4-point scale (1=not representative or relevant or clear to 4=representative or relevant or clear). In addition, experts will be invited to provide qualitative feedback on the items, instructions, and rating scales, including written comments to justify their ratings and suggest revisions. Finally, given the amount of material to be assessed and the estimated completion time of approximately 75 minutes, experts will be invited to complete the review at their own pace and may take breaks as needed before submitting their evaluation.

#### Analysis

The content validity index (CVI) will be calculated to examine the proportion of agreement between the experts for each item. The literature suggests eliminating items with a CVI below 0.70 and modifying items scoring between 0.70 and 0.79 [84,87]. A CVI of 0.80 or higher is considered acceptable [84,87]. Additionally, an overall CVI score will be calculated to measure the overall agreement on the items. For a new assessment tool, an overall CVI of at least 0.80 is recommended [87]. Experts will also be invited to assess the overall comprehensiveness of the assessment tool and to propose modifications to enhance the representativeness, relevance, and clarity of the items [84]. Finally, qualitative feedback regarding the items, instructions, and rating scales will be analyzed according to a thematic analysis strategy (see Phase 3 for a detailed description) [90]. Decisions concerning the retention, elimination, or modification of items, instructions, or rating scales will be established by considering both quantitative and qualitative data. A revised version that considers the results obtained at this stage will be submitted for a pretest.

### Phase 3 – Pretest

A pretest will be conducted to pursue two main objectives: (1) to ensure that the tool is well understood and appropriately interpreted by its intended users [91-93], and (2) to assess its acceptability, applicability, and transferability [94-96]. Based on cognitive interviewing, this phase will help identify potential shortcomings in the tool that could lead to response errors among users [91,97-100]. Moreover, including an assessment of applicability and transferability at this stage allows for a comprehensive understanding of both the tool itself and the conditions under which it can be effectively used across various organizational settings, which is particularly relevant for public health initiatives such as this one [82,94].

#### Participants

Five participants representing the tool’s intended users [92] will be recruited using a purposive sampling strategy. This sample size is consistent with recommendations for cognitive interviewing studies. Additional participants and/or an additional round of approximately 5 participants may be included if needed to ensure sufficient informational redundancy to meet the objectives of this phase. Inclusion criteria for this phase are: (1) having at least 2 years of professional experience in a health and social services organization in Quebec, (2) working in tasks related to the prevention of occupational injuries or prolonged incapacity at work, and (3) being involved in prevention efforts intended for workers within the health and social services organizations in Quebec.

#### Procedure

Participants will be invited to complete the assessment tool based on the reality of their own organizational context. Their perceptions will then be gathered through individual semistructured interviews. An interview guide, which will undergo its own pretest, containing open-ended questions will be used [92,93]. Interview questions will be developed by using cognitive interviewing techniques such as verbal probing (eg, general and specific probes) [99,100], or key indicators of acceptability, applicability, and transferability [94,101]. More specifically, the questions will address 4 key themes related to the tool: (1) clarity (eg, what came to mind when you read those less-clear items?), (2) acceptability (eg, tell me about your satisfaction with the effectiveness of the assessment tool in measuring the characteristics of integrative prevention at work within health and social services organizations?*),* (3) applicability (eg, tell me about the resources available in your organizational context that could support the use of this assessment tool), and (4) transferability (eg, tell me about the contextual elements that could influence the use of the tool and the results obtained from it). Finally, a general question will be asked to allow participants to add clarifications or any additional elements not covered during the interview (eg, do you have any other elements you would like to share regarding the content, administration, or any other aspect of the evaluation tool?).

#### Analysis

Data emerging from the interviews will be analyzed using a thematic analysis strategy [90]. This analysis will be conducted in six iterative steps by 2 members of the research team, following the approach proposed by Braun and Clarke [90]: (1) familiarization with the data, (2) generating initial codes, (3) searching for themes, (4) reviewing themes, (5) defining and naming themes, and (6) producing the report. The analysis of the 5 interviews will follow a rigorous validation process. Two researchers (AAD and to be confirmed) will independently perform each analytic step before jointly reviewing their coding to achieve consensus on the resulting codes and themes. A third member of the team (AL) will subsequently assess the unified coding framework to ensure consistency. Such triangulation within the research team helps minimize the risk of bias by preventing the coding process from reflecting the perspective of a single individual [102]. Furthermore, an audit trail of code generation will be maintained, documenting the development and refinement of codes and themes to enhance the overall quality of the analysis [102]. The analysis will be supported using QDA Miner 5 software (Provalis Research). Finally, at the end of the data analysis, the assessment tool is expected to contain at least 15 items (ie, 3 items per each of the 5 subscales) to proceed with the evaluation of its psychometric properties [80,103].

### Phase 4 – Evaluation

Psychometric properties of the tool, namely factorial validity and internal consistency, will be evaluated.

#### Participants

People working in health and social services organizations in Quebec who meet the inclusion and exclusion criteria described in phase 3 will be invited to participate. The exact number of participants will be determined at this phase, as it depends on factors such as the number of items per factor and item communalities (eg, [104,105]). Nonetheless, the literature recommends recruiting a minimum of 5 participants per item [106-108]. For example, if the final version of the assessment tool includes 15 items at the end of phase 3, at least 75 participants will be recruited for phase 4.

#### Statistical Analysis

Before conducting any analyses, data normality will be examined using histograms, skewness, and kurtosis indices, and visual inspection of Q-Q plots, boxplots, and stem-and-leaf plots [109,110]. Bartlett test of sphericity and the Kaiser-Meyer-Olkin (KMO) measure of sampling adequacy will be performed to assess the suitability of the data for factor analysis [109-111]. The responses will be treated as approximately continuous in the exploratory and confirmatory factor analyses. This approach is supported by methodological recommendations indicating that ordinal variables with 7 response categories can be treated as approximately continuous when their distributions are adequate [112]. To evaluate factorability, an exploratory factor analysis will first be conducted using the principal axis factoring method [109,113]. An oblique rotation will be applied, as the factors of this assessment tool are expected to be correlated, for example, communication and collaboration between stakeholders [49]. This situation is commonly the case in social science scales [114]. To determine the optimal number of factors to retain, parallel analysis [115] and the scree test [116] will both be used [108,117]. Afterwards, the most valid factor solution will be tested using confirmatory factor analysis, following the principles of structural equation modeling [118]. Several fit indices will be examined to assess the overall model fit, including the chi-square test (with its degrees of freedom and *P* value), the Tucker-Lewis index (TLI), the comparative fit index (CFI), the standardized root mean square residual (SRMR), and the root mean square error of approximation (RMSEA), along with its CI and *P* value (ie, pclose) [110,111,113,118,119]. To evaluate internal consistency, the McDonald omega coefficient will be calculated [120,121]. This method is chosen over Cronbach α due to its greater flexibility and its ability to account for the multifactorial nature of the data, which will most likely reflect the structure of our dataset [120,122,123]. A value greater than 0.70 is considered acceptable [120]. Statistical analyses will be performed using SPSS software (version 29.0; IBM Corp), along with its Analysis of Moment Structures (AMOS) extension, with the support of a statistician.

### Ethical Considerations

The study received ethical approval from the Human Research Ethics Committee of the University of Quebec in Trois-Rivières (CER-25-320-07.14).

## Results

This study will offer a new validated tool to assess the attributes of integrative prevention at work. At the time of manuscript submission in January 2026, phase 1 had been completed.

In this first phase, a preliminary version of the assessment tool was developed. The initial 96 items were distributed as follows: 25 items for holistic vision of health, 11 for common understanding of the purpose of integrative prevention at work, 20 for communication between stakeholders, 19 for collaboration between stakeholders, and 21 for coordination of preventive actions. For example, to assess the holistic vision of health, the following item is proposed: “In my organization, relevant stakeholders actively promote healthy behaviors at work*.*” Regarding the coordination of preventive actions, the assessment tool includes the following item: “In my organization, relevant stakeholders provide preventive actions that are complementary.” As for communication between stakeholders, this item is proposed: “In my organization, relevant stakeholders explicitly encourage workers to express their concerns regarding their health, safety, and well-being.” These items are accompanied by instructions asking respondents to rate the frequency with which each attribute of the approach is manifested within the organization where they work or with which they collaborate. Responses are provided on a 7-point ordinal scale ranging from “never” to “always.” This preliminary version of the assessment tool will be submitted to phase 2.

Recruitment for phase 2 is currently underway at the time of manuscript submission. A separate peer-reviewed publication presenting the detailed results from phases 1 and 2 is planned for submission by the end of 2026. Additional publications related to phases 3 and 4 are also planned in the coming years and are expected by the end of 2028.

## Discussion

### Overview

This is the first protocol to operationalize the recently conceptualized approach of integrative prevention at work through the development of a validated assessment tool. Several findings are expected and will contribute to the advancement of knowledge from organizational and research perspectives. Future directions are also discussed, as well as study strengths and limitations.

### Expected Findings

From an organizational perspective, this research project will provide the first assessment tool capable of evaluating all the attributes of integrative prevention at work on a unified scale. It will also be the first tool aligned with the most recent conceptualization of integrative prevention at work. Building on gaps identified in the existing literature, this tool uniquely integrates the 5 attributes of integrative prevention at work within a single assessment tool [51]. In addition, the psychometric properties of the tool will be evaluated and made available, an aspect rarely reported for comparable tools [51]. The tool offers organizations a simple, contextually adaptable resource that allows them to initiate necessary changes to implement an integrative prevention at work approach and to more accurately identify their strengths and weaknesses in prevention practices. Its development is grounded in the recognition that previous initiatives in the health and social services sector, namely PEARS in Canada and LWWW in the United States, have highlighted the relevance and potential benefits of integrative prevention approaches, as well as the sector’s growing interest in such approaches. This tool provides a structured way to assess and monitor these kinds of approaches, supporting organizations in their adoption and ongoing refinement, thereby helping to address some implementation challenges observed in those initiatives. In fact, given the dynamic nature of integrative prevention at work, the assessment tool may be administered at multiple time points to monitor implementation progress and changes in prevention practices over time. In the long term, this tool is expected to support the development of comprehensive and coordinated prevention practices within organizations, facilitating the transition from isolated initiatives to integrated approaches. Implementation initially focuses on health and social services organizations in Quebec, where the needs for integrative prevention approaches are particularly pressing. However, the tool also has potential for adaptation and application in other health and social services sectors nationally and internationally, as well as in other sectors of activity, providing a scalable resource for diverse organizations. In other words, this assessment tool represents a concrete and innovative deliverable that can be readily used by stakeholders to facilitate the implementation and monitoring of a promising prevention approach. The 5 attributes will initially be interpreted separately to examine the frequency with which each attribute is manifested within an organizational context. Taken together, the 5 attributes may provide an overall understanding of the frequency with which integrative prevention at work is manifested within an organization. However, a single overall score will not be assumed unless its theoretical and empirical justification is supported by the psychometric evaluation. If supported, such a score would be intended to inform continuous improvement within each organization. The assessment tool is therefore intended primarily to support reflection and the development of better preventive practices. As similar tools have demonstrated, we expect that this tool will facilitate communication and collaboration between stakeholders [124-126], and even empower them by enhancing their agency and control over preventive practices in their workplaces [125,127].

From a research perspective, this study contributes to advancing knowledge on the prevention continuum in occupational health. Specifically, it is expected to generate empirical evidence supporting the integrative prevention at work approach and to deepen our understanding of its implementation. In doing so, it will clarify key conceptual dimensions, respond to the need for empirical data highlighted by the scientific community [40], and thus provide a methodological foundation for future studies. Furthermore, the development and validation of this tool address a gap previously identified in the literature [41,51].

### Future Directions and Research Dissemination

This project will offer an operational tool to facilitate the implementation of integrative prevention at work and to assess the frequency with which its 5 attributes are manifested within the health and social services organizations. The tool could serve as a foundation for the development of a training workshop focused on its use. This workshop would be intended for its target users, namely professionals working in health and social services organizations who are involved in tasks related to the prevention of occupational injuries or the support of return-to-work processes for workers who have experienced a period of absence in this sector. To support the dissemination of findings and usage guidelines, we also plan to develop a summary document highlighting key information, which will be distributed throughout the health and social services sector in Quebec, Canada. Partnerships with various occupational health organizations and key stakeholders (eg, decision-makers in the targeted sector) are also being pursued to facilitate the implementation of this tool in organizational settings.

We also plan to disseminate content through social media and participate in podcasts to communicate our findings to a broader audience. In particular, a blog dedicated to various themes related to integrative prevention at work will be developed and promoted across different digital platforms. By using a variety of accessible and engaging knowledge translation formats tailored to diverse preferences, we hope to reach a broader audience while accounting for individual preferences and diversity.

For the scientific community, through the publication of open-access scientific papers, as well as participation in conferences, this project will provide empirical evidence on integrative prevention at work. This research project will also pave the way for future research. Additional psychometric properties could be explored in future studies (eg, test-retest reliability). Moreover, the project could lead to the transcultural validation of the tool in English or other languages, expanding its applicability to contexts beyond the one outlined here.

### Strengths and Limitations

To our knowledge, this study presents the protocol for the first assessment tool capable of evaluating all 5 attributes of integrative prevention at work using a unified scale. The development and validation of such a tool will fill a gap in the current literature regarding the need for a tool to operationalize this concept within organizations [14,51]. The methodological study is described in detail to ensure scientific rigor and replicability in future studies [77]. The validation processes outlined in this protocol aim to provide a tool tailored to organizations in the health and social services sector in Quebec, Canada, offering them a concrete and practical deliverable. Nevertheless, the transferability of the results to other contexts cannot be guaranteed. Further methodological limitations can be anticipated, one of which concerns the sample size in phase 4, required for conducting factorial analysis. Although we plan to follow the recommended ratio of 5 participants per item [106-108,111], this remains a minimal threshold. As noted in the methodological section, our sample size could be as small as 75 participants. However, several authors recommend not only considering the participant-to-item ratio, but also ensuring a minimum sample size of 100 participants [117], or even between 200 and 500 participants for more robust and reliable results [128]. We aim to recruit as many participants as possible to meet these higher thresholds. However, for feasibility reasons, we may only be able to meet the minimal recommended ratio. While this would not invalidate the results, since many published studies have conducted factorial analyses with relatively small samples [108], it would nonetheless be advisable to replicate these analyses with larger samples in future research in order to draw stronger conclusions. Moreover, because this manuscript presents a study protocol, certain analytical decisions will be informed by the results of the psychometric evaluation. For example, the calculation of a potential overall score will depend on the factorial structure identified during phase 4 and on whether such a score is theoretically and empirically justified. Similarly, procedures for handling missing data will be selected in accordance with methodological recommendations and the psychometric characteristics of the final version of the assessment tool. Finally, a limitation of the assessment tool is that it focuses on the 5 defining attributes of integrative prevention at work and therefore does not provide a comprehensive assessment of the entire conceptual model, including its antecedents, outcomes, and contextual factors. However, it provides a starting point for assessing the frequency with which attributes of integrative prevention at work are manifested within organizations.

### Conclusions

This paper presents a research protocol designed to develop and validate a tool for assessing the 5 attributes of integrative prevention at work, using a 4-phase methodological study design. This tool will support the operationalization of integrative prevention in health and social services organizations in Quebec, Canada. The project is expected to generate multiple benefits from clinical, organizational, societal, and research perspectives. By providing organizations and stakeholders with a concrete means to evaluate and strengthen their preventive practices, this tool may foster a cultural shift toward more sustainable and integrative approaches to occupational health. In addition to contributing to the advancement of knowledge in the field of the continuum of prevention for occupational injuries and prolonged incapacity at work, the tool could also serve as a model for future tool developments in other sectors, thereby promoting the broader adoption of integrative prevention approaches.

## Abbreviations

AMOS: Analysis of Moment Structures
CFI: comparative fit index
CVI: content validity index
KMO: Kaiser-Meyer-Olkin
LWWW: live well/work well
PEARS: Prevention and Early Active Return-to-Work Safely
RMSEA: root mean square error of approximation
SRMR: standardized root mean square residual
TLI: Tucker-Lewis Index
TWH: total worker health
